# Supplementary material for: Cryo-EM structure of ssDNA bacteriophage ΦCjT23 provides insight into early virus evolution
Source: Nat Commun. 2022 Dec 3;13:7478. doi: 10.1038/s41467-022-35123-6 (PMC9719478; doi:10.1038/s41467-022-35123-6)

Supplementary information to:

Cryo-EM structure of ssDNA bacteriophage ΦCjT23 provides insight into early virus evolution

Nejc Kejzar[1,#,§], Elina Laanto[2,3,#], Ilona Rissanen[1,#], Vahid Abrishami[1], Muniyandi Selvaraj[1,¶], Sylvain Moineau[4], Janne Ravantti[2], Lotta-Riina Sundberg[3,*], Juha T. Huiskonen[1,*]

[1] Institute of Biotechnology, Helsinki Institute of Life Science HiLIFE, University of Helsinki, Finland
[2] Molecular and Integrative Biosciences Research Programme, Faculty of Biological and Environmental Sciences, University of Helsinki, Finland
[3] Department of Biological and Environmental Science, Nanoscience Center, University of Jyväskylä, Finland
[4] Université Laval, Département de biochimie, de microbiologie, et de bio-informatique, Québec City, Québec, Canada

[§] current address: Krupic lab, Department of Physiology, Development and Neuroscience, University of Cambridge, Cambridge CB2 3EG, UK
[¶] current address: The Sainsbury Laboratory, Norwich Research Park, Norwich NR4 7UH, UK

[*] corresponding authors (juha.huiskonen@helsinki.fi; lotta-riina.sundberg@jyu.fi)
[#] contributed equally

Supplementary Tables

Supplementary Table 1 | ΦCjT23 cryo-EM data collection and processing details.

	**Virion** (EMD-15042) (PDB 7ZZZ)	**Trimer 1** EMD-15044) (PDB 8A01)	**Trimer 2** EMD-15045) (PDB 8A02)	**Trimer 3** (EMD-15046) (PDB 8A03)	**Trimer 4** EMD-15047) (PDB 8A04)	**Spike, penton domain** (EMD-15048) (PDB 8A05)	**Spike, external part** (EMD-15049)
**Data collection and processing**							
Magnification	120,000×	120,000×	120,000×	120,000×	120,000×	120,000×	120,000×
Voltage (kV)	200	200	200	200	200	200	200
Electron exposure (e^{-}/Å^2)	15	15	15	15	15	15	15
Defocus range (μm)	0.3–2.2	0.3–2.2	0.3–2.2	0.3–2.2	0.3–2.2	0.3–2.2	0.3–2.2
Pixel size (Å)	1.24	1.24	1.24	1.24	1.24	1.24	1.24
Symmetry imposed	I1	C1	C1	C3	C1	C5	C5
Initial particle images (no.)	8,862	390,060	390,060	130,020	390,060	78,012	74,668
Final particle images (no.)	6,501	265,790	257,197	98,641	265,085	18,122	40,397
Map resolution (Å)	4.1	3.2	3.2	3.2	3.2	3.4	5.3
FSC threshold	0.143	0.143	0.143	0.143	0.413	0.143	0.143
Map sharpening *B* factor (Å^2)	–151	–141	–156	–139	–150	–151	–219
**Refinement & validation**							
Model-to-map resolution (Å)	4.3	3.3	3.4	3.4	3.4	3.8	N/A
FSC threshold	0.5	0.5	0.5	0.5	0.5	0.5	N/A
Model-to-map CC							
Main chain	0.82	0.86	0.84	0.84	0.85	0.83	N/A
Side chain	0.81	0.83	0.82	0.82	0.83	0.81	N/A
Model composition							
Non-hydrogen atoms	19134	5511	5511	5511	5511	764	N/A
Protein	19134	5511	5511	5511	5511	764	N/A
Ligand	0	0	0	0	0	0	N/A
Model resolution range (Å)	N/A	3.2	3.2	3.2	3.2	3.4	N/A
B factors (Å^2)							
Protein	N/A	33.5	31.9	45.6	34.3	45.1	N/A
R.m.s. deviations							
Bond lengths (Å)	N/A	0.002	0.003	0.002	0.004	0.002	N/A
Bond angles (°)	N/A	0.439	0.469	0.437	0.599	0.424	N/A
Validation							
MolProbity score	N/A	1.25	1.21	1.21	1.21	1.01	N/A
Clash score	N/A	4.75	4.31	3.41	4.31	1.99	N/A
Rotamer outliers (%)	N/A	0	0	0	0	0	N/A
Ramachandran plot							
Favored (%)	N/A	98.45	98.03	97.61	98.45	97.83	N/A
Allowed (%)	N/A	1.55	1.97	2.39	1.55	2.17	N/A
Outliers (%)	N/A	0	0	0	0	0	N/A

Supplementary Table 2 | FLiP cryo-EM data processing details.

	**Penton** (EMD-15051) (PDB 8A06)
**Data processing**	
Pixel size (Å)	1.35
Symmetry imposed	C5
Initial particle images (no.)	141,060
Final particle images (no.)	28,212
Map resolution (Å)	4.0
FSC threshold	0.143
Map sharpening *B* factor ($Å^2$)	–164
**Refinement**	
Model-to-map resolution (Å)	4.3
FSC threshold	0.5
Model-to-map CC	
Main chain	0.75
Side chain	0.70
Model composition	
Non-hydrogen atoms	1185
Protein	1185
Ligands	0
Model resolution range (Å)	4.0
B factors ($Å^2$)	
Protein	36.2
R.m.s. deviations	
Bond lengths (Å)	0.003
Bond angles (°)	0.556
Validation	
MolProbity score	1.47
Clash score	5.94
Rotamer outliers (%)	0.73
Ramachandran plot	
Favored (%)	97.24
Allowed (%)	2.76
Outliers (%)	0

Supplementary Figure Legends

Supplementary Figure 1 | Comparison of detected ΦCjT23-like prophages. An Easyfig alignment of the representative prophage regions in *Flavobacterium* and *Lacinutrix* sp (*Flavobacteriaceae)* genomes. Genes with putative functions are marked with colors with indicating functions shown in the bottom. Some of the prophage genes received hits in a BlastP search against ssDNA phages FLiP (all hits to FLiP gp16) and Cellulophaga phages phi48:2, phi12:a:1, phi12:2 and phi18:4, and these hits are also indicated with colors in the bottom. In addition, several of the replication proteins showed similarities with the small Cellulophaga ssDNA phages. Also, the identified major capsid protein sequence showed similarities with the structural proteins of these phages.

Supplementary Figure 2 | Cryo-EM and three-dimensional reconstruction of ΦCjT23 bacteriophage and its capsid components. (a) A representative cryo-EM micrograph of purified ΦCjT23 particles. One smaller particle, possibly representing the lipid core of ΦCjT23, is marked with an asterisk. There were 1023 micrographs in total, most with similar results. **(b)** Representative class averages of particles. One class average, representing the putative lipid core, is marked with an asterisk. **(c)** A 5-Å thick central cross-section of the conventional icosahedral reconstruction. DNA and membrane (M) densities are labelled. The inset shows a close-up (3×) of one spike (arrowhead). **(d)** Areas for different localized reconstructions are illustrated with colored transparent spheres on the icosahedral reconstruction (gray). Note that for clarity, the spheres are rendered smaller than the localized reconstructions. The insets show resulting localized reconstructions of the spike (purple) and vertex (cyan) are shown from side. **(e)** The localized reconstructions of the trimers 1–4 are shown from the top. **(f)** A 5-Å thick central section of a composite volume of the particle (EMD-15050), calculated by combining the localized reconstructions. The inset shows a close-up (3×) of one spike (arrowhead) with improved density over the conventional map (*c*, inset). Scale bars, 50 nm. **(g)** Comparison of ΦCjT23 spike external part to FLiP spike (EMD-3771, filtered to 5.3-Å resolution).

Supplementary Figure 3 | Resolution and map quality of the major capsid protein. (a) Fourier shell correlation plots (FSC), calculated between two half-maps as a function of spatial frequency, are shown for each trimer (1–4) localized reconstruction. In each case, FSC is plotted for the original unmasked half-maps (gray), masked half-maps (blue), and phase-randomized half-maps (red) in which phases were randomized at frequencies higher than 1/7 Å. The phase-randomized FSC drops sharply at the cutoff frequency below the noise threshold (0.143), as expected. The phase-randomization test was used to take the effect of masking on the half-maps into account before calculating the final, corrected FSC curve (black). Good agreement between the masked and corrected curves indicated that masking did not cause overestimation of resolution. In all cases, the corrected curve drops below the noise threshold (FSC=0.143) at 1/3.2 Å indicating a resolution of 3.2 Å in the reconstructions. **(b–d)** Selected parts of the trimer 3 map V1 domain are shown, namely the FG1 α-helix (*b*), β-sheet C-H-E-F (*c*) and β-sheet B-I-D-G (*d*). Source data are provided as a Source Data file.

Supplementary Figure 4 | Major capsid protein antiparallel β-sandwich structures. Different structural elements are shown as topology diagrams for the N-terminal (V1) and C-terminal (V2) β-sandwich with arrows (β-strands), boxes (α-helices) and arcs (linker elements between β-strands and termini). β-strands are labeled B1–I1 in V1 and B2–I2 in V2. Linkers consist of β-turns or longer loops. The approximate length of each linker element is given. Longer linkers contain in some cases additional secondary structure elements. Short secondary structure elements (four residues or shorter) are not shown for clarity. α-helices residing on the membrane-proximal side of the β-sandwich in FLiP, PM2 and PRD1 are colored in red. The insets show the corresponding ribbon representation for each domain. The same elements are highlighted in red as in the topological diagrams.

Supplementary Figure 5 | Comparison of MCPs chains. (a) All ten major capsid protein chains from the asymmetric unit (three for trimers 1,2 and 4; one for trimer 3) are shown after aligning them. The two β-sandwiches V1 and V2 are labeled. (**b**) The view in *a* is shown after rotating 90 degrees around the horizontal axis as indicated to view the monomer from outside of the capsid. Note that little structural variation exists in the FG1 and FG2 loops (labeled). (**c**) The view in *a* is shown after rotating 90 degrees around the horizontal axis as indicated to view the monomer from inside of the capsid. The two loops showing the greatest degree of structural variation, EF1 and GH1 loop, are labeled. **(d)** A close-up of the EF1 loops is shown for the area indicated in (*c*). The three discrete conformations are labeled (outward, middle, inward). The arc depicts the range of motions this loop can undergo. **(e)** All chains in our asymmetric

unit (colored as in Figure 1) are shown, together with their neighbors (gray). The type of each EF1-loop is indicated (outward, O; middle, M; inward I). The demarcation lines between the icosahedral faces are shown with dashed lines. **(f)** Examples for each EF1-loop type are shown from the inside of the capsid. The trimer the EF1-loop interacts with is shown as a surface.

Supplementary Figure 6 | Resolution and map quality of the ΦCjT23 penton domain and FLiP penton protein. **(a)** Fourier shell correlation (FSC) is shown for the ΦCjT23 penton domain localized reconstruction. Different curves are as in Supplementary Figure 3a. The corrected curve drops below the noise threshold (FSC=0.143) at 1/3.4 Å indicating a resolution of 3.4 Å in the reconstruction. **(b–c)** Examples of the ΦCjT23 penton domain cryo-EM density are shown for an α-helix (*b*) and a β-sheet (*c*). **(d)** FSC is shown for the FLiP penton protein localized reconstruction. The corrected curve drops below the noise threshold at 1/4.0 Å indicating a resolution of 4.0 Å in the reconstruction. **(e–f)** Examples of the FLiP penton protein cryo-EM density are shown for an α-helix (*e*) and a β-sheet (*f*). Source data are provided as a Source Data file.

Supplementary Figure 7 | The ΦCjT23 penton domain has a distinct fold. **(a)** The common penton structure of the PRD1–adenovirus lineage, represented by PRD1 penton protein (PDB:1W8X), is shown. **(b)** The same topology is observed in the FLiP penton. **(c)** ΦCjT23 capsid internal part of the spike (penton domain) shows a distinct, unrelated fold. For each penton structure, a ribbon representation, colored rainbow ramped from blue (N-terminus) to red (C-terminus), is shown (left) alongside a topology diagram (right). Secondary structure elements in the topology diagram are presented with arrows (β-strands) and cylinders (α-helices) and are colored as in the respective ribbon representations.

Supplementary Figure 8 | Adsorption of ΦCjT23 to the cells of *Flavobacterium*. Dots represent individual values of three replicates, horizontal line their mean, and vertical line standard error. Source data are provided as a Source Data file.

Supplementary Figure 1

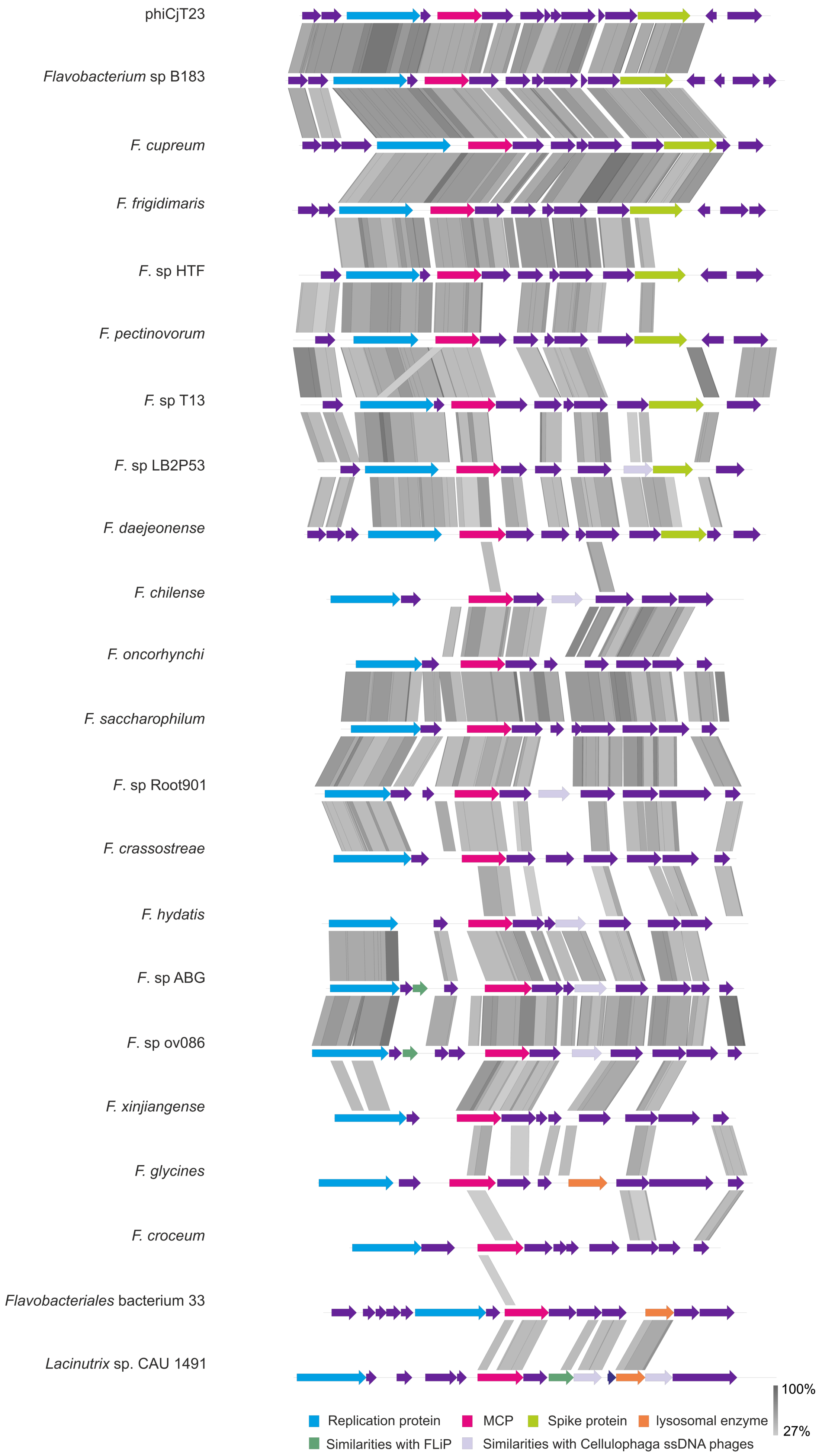

Supplementary Figure 2

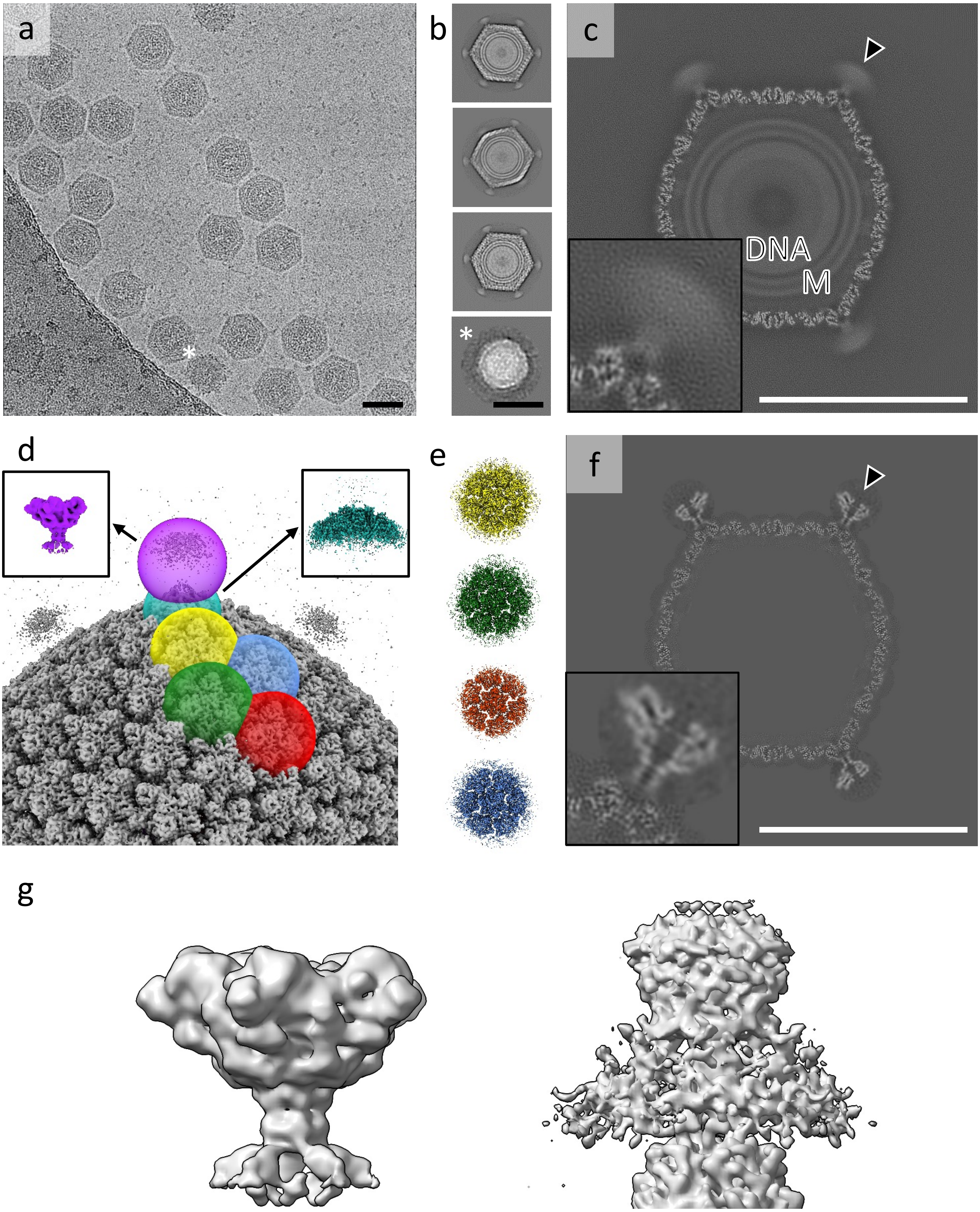

phiCjT23 spike
(external part)

FLiP spike

Supplementary Figure 3

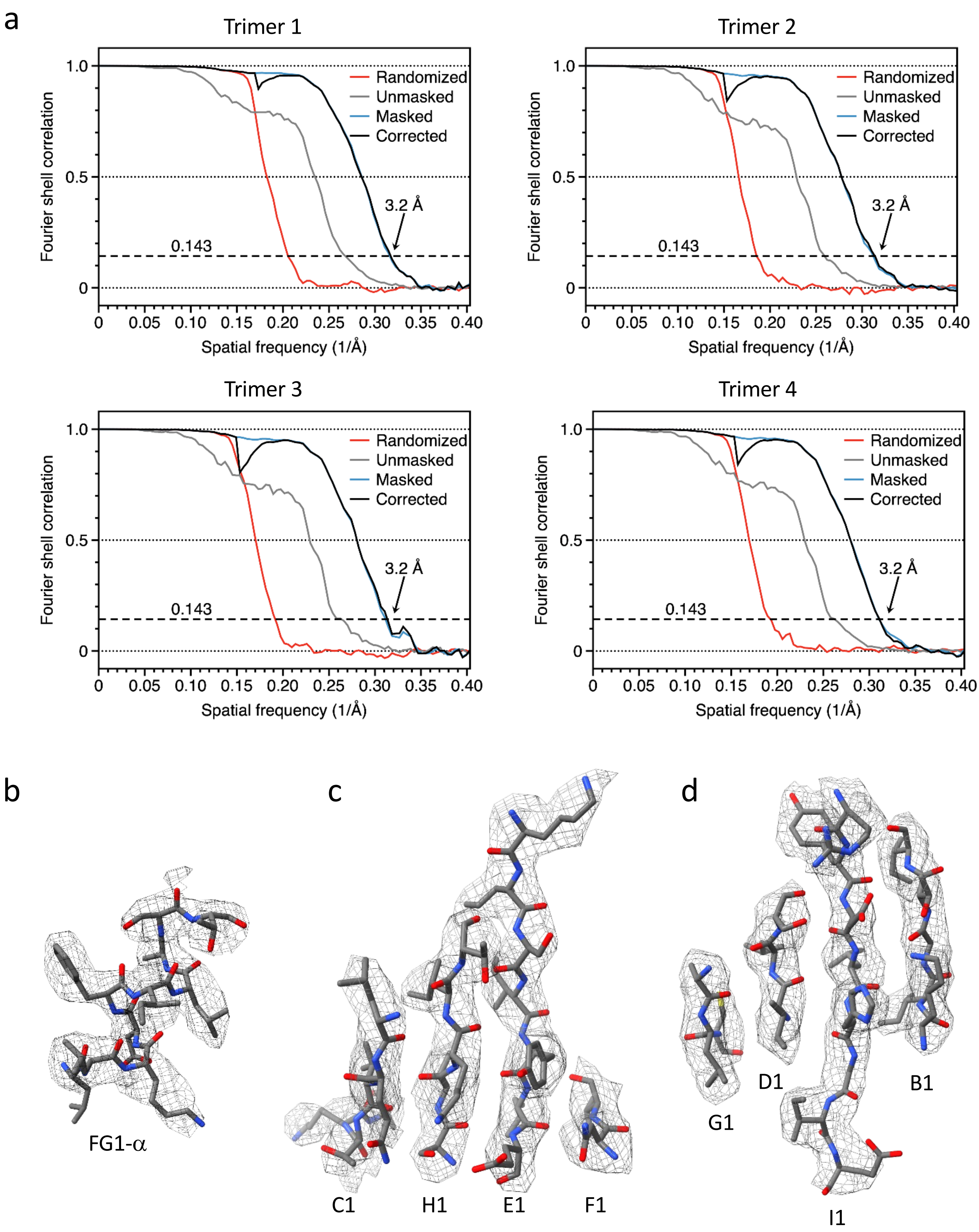

Supplementary Figure 4

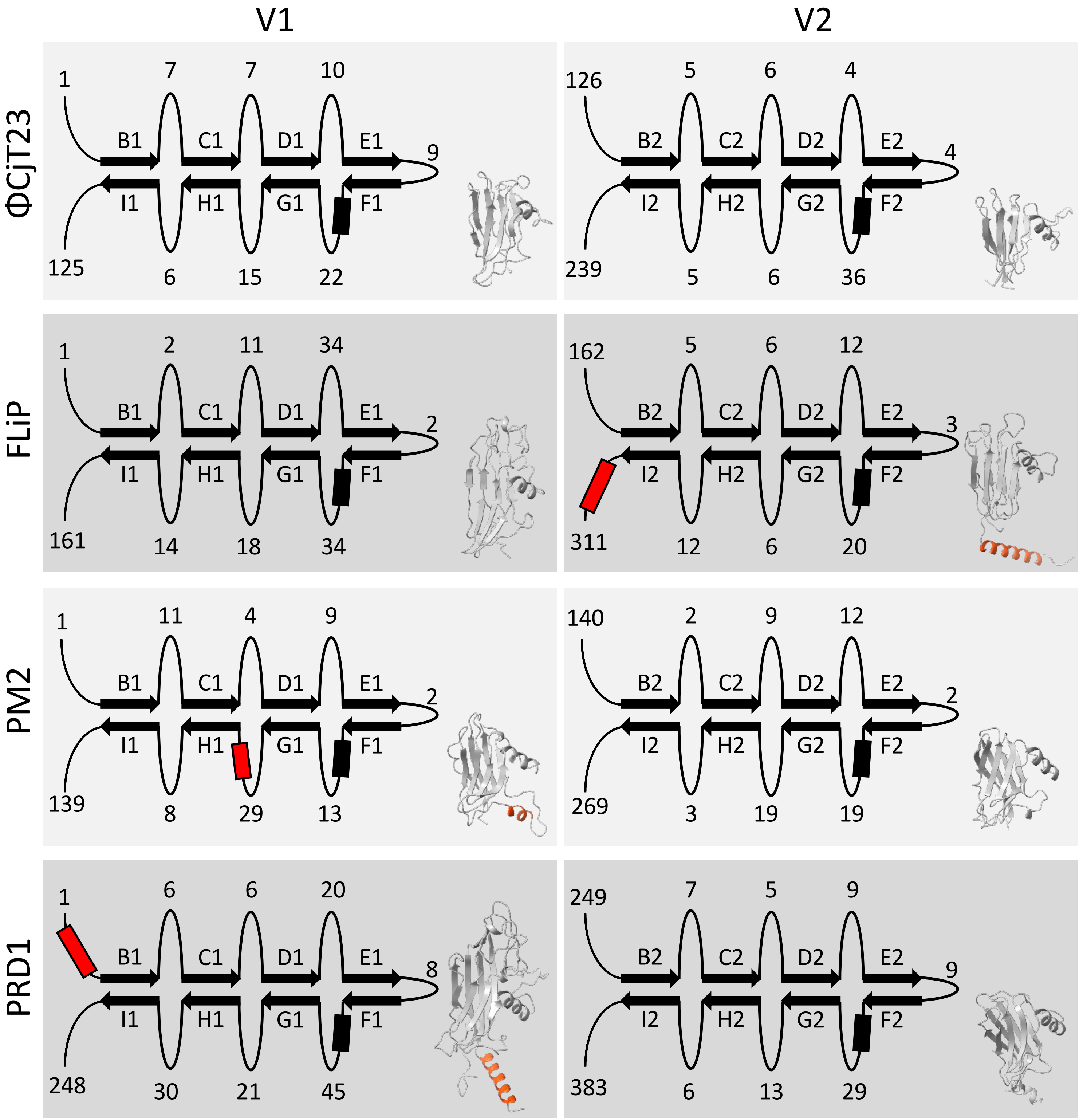

Supplementary Figure 5

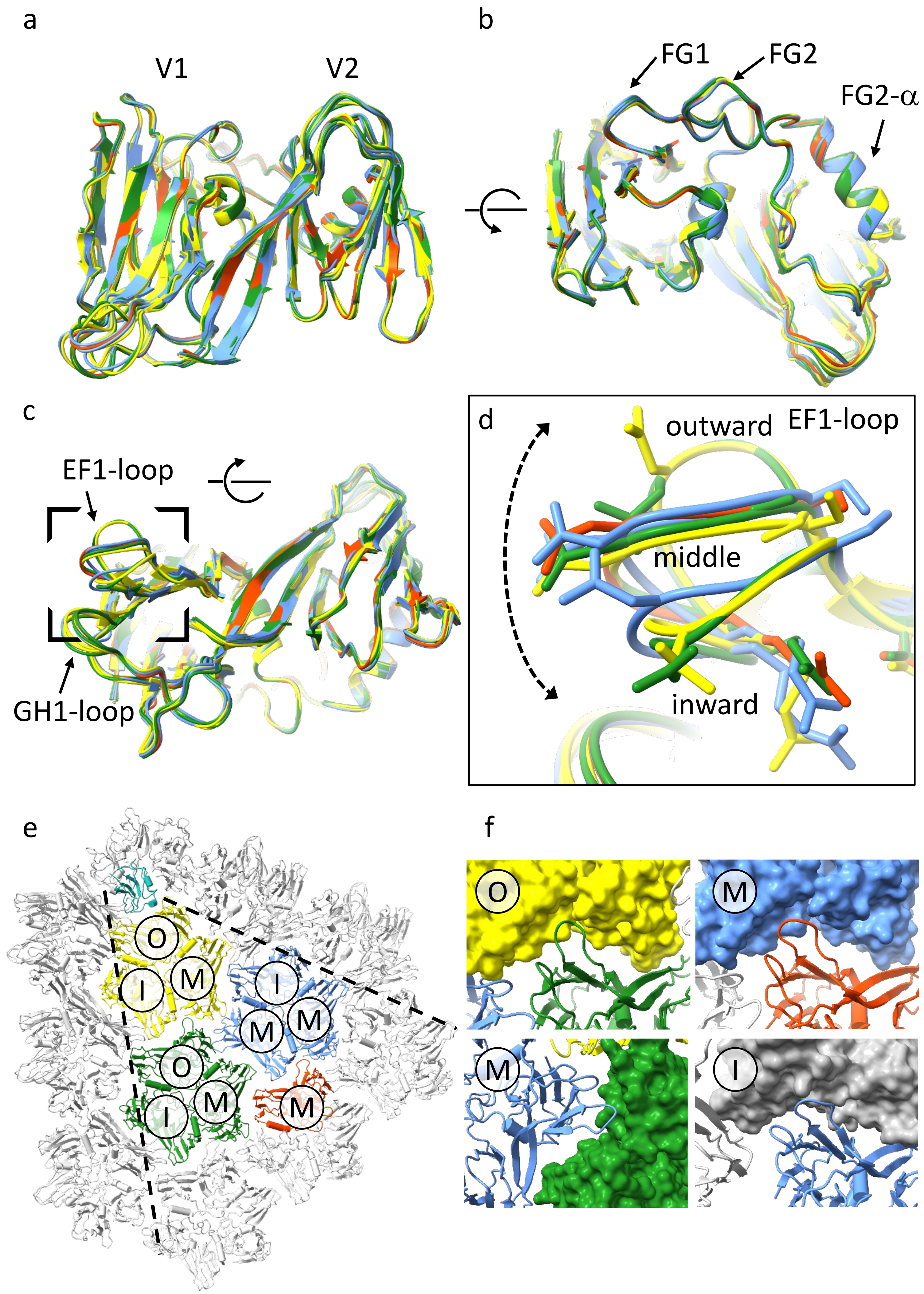

Supplementary Figure 6

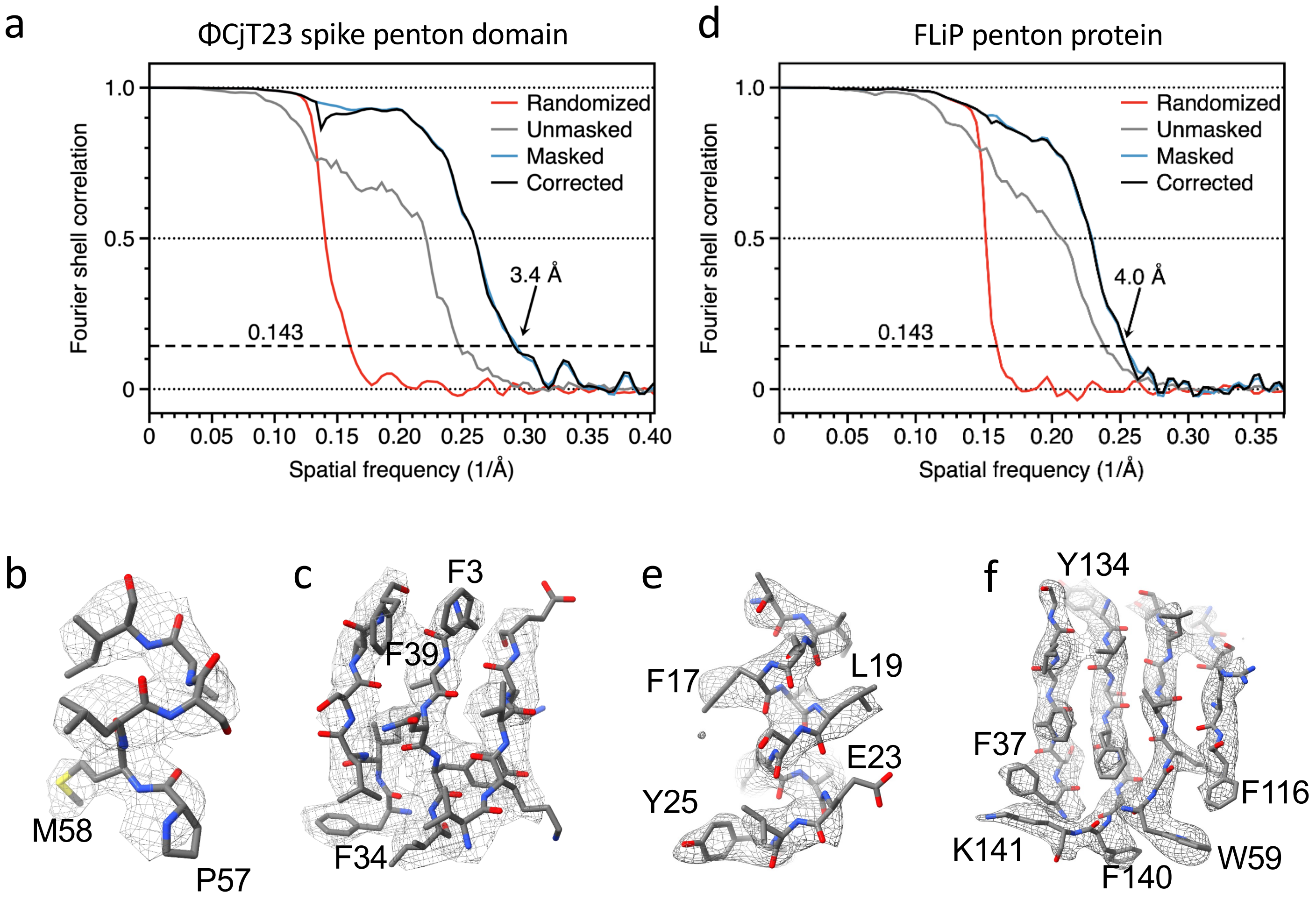

Supplementary Figure 7

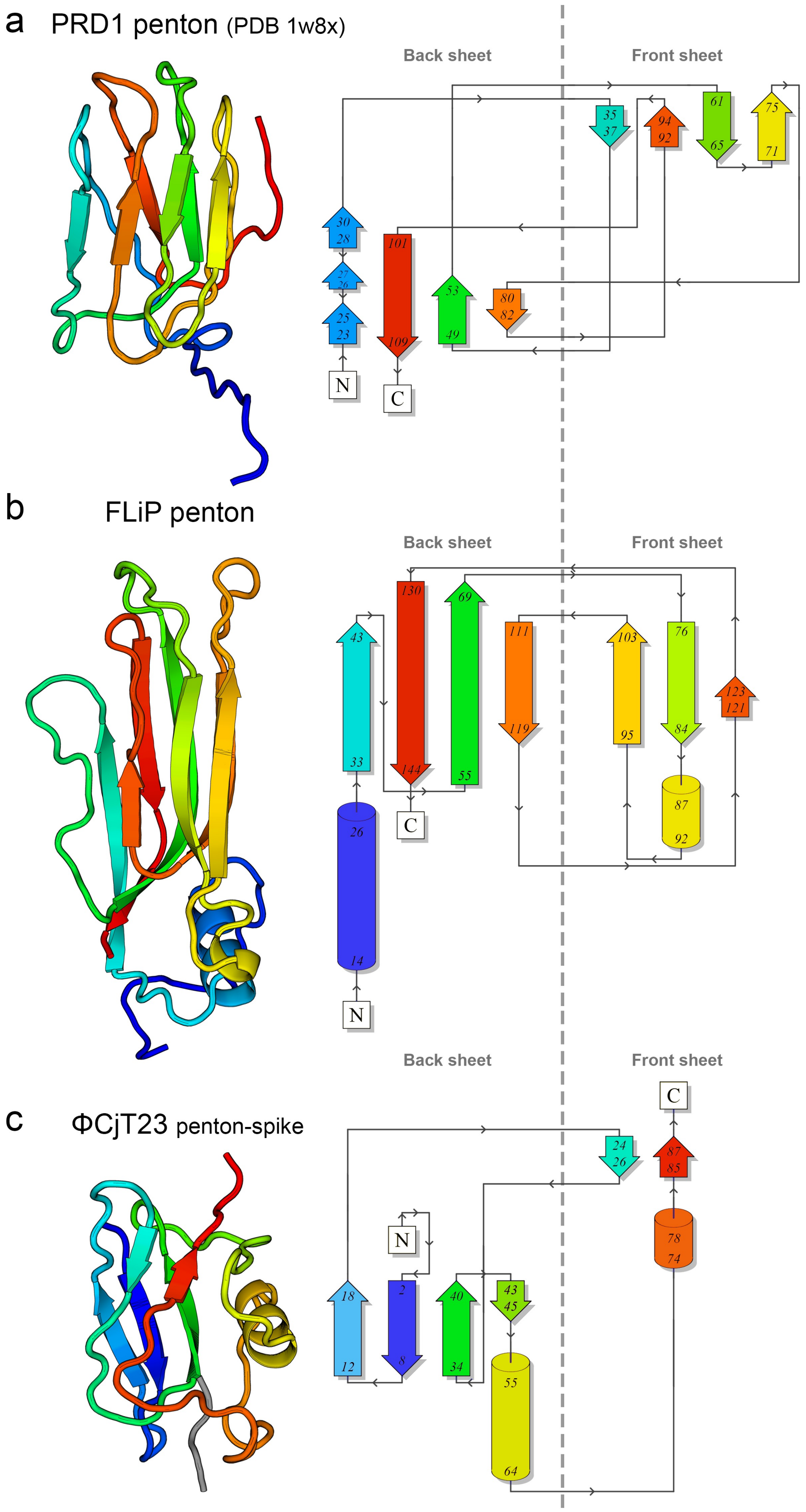

Supplementary Figure 8

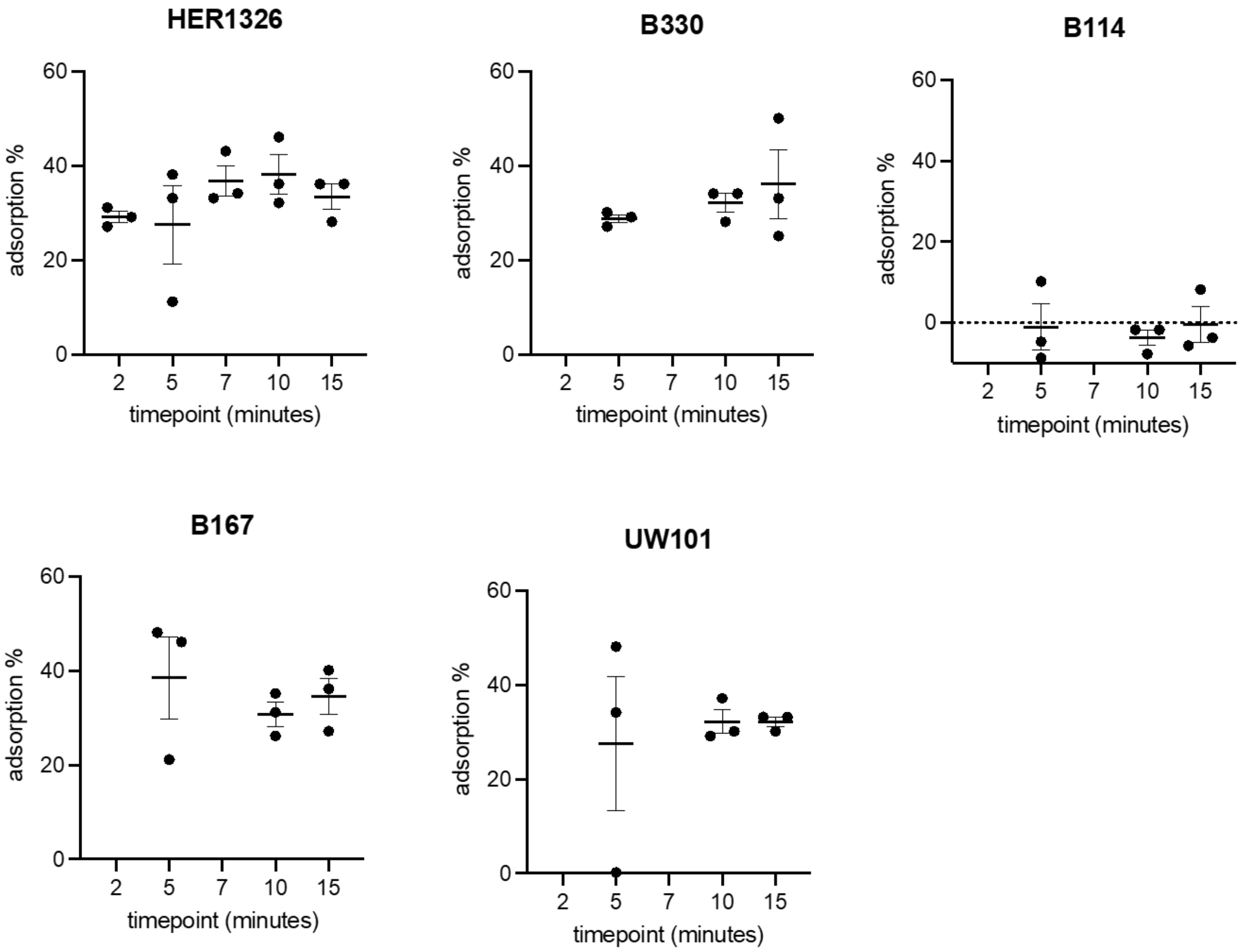

Supplement: Supplementary file 1 — Supplementary Information [file 41467_2022_35123_MOESM1_ESM.pdf]
