# Supplementary material for: Cryo-EM structure of ssDNA bacteriophage ΦCjT23 provides insight into early virus evolution
Source: Nat Commun. 2022 Dec 3;13:7478. doi: 10.1038/s41467-022-35123-6 (PMC9719478; doi:10.1038/s41467-022-35123-6)
Supplement: Supplementary file 2 — Description of Additional Supplementary Files [file 41467_2022_35123_MOESM2_ESM.pdf]

Title: Supplementary Data 1:
Description: phiCjT23 genome annotation

Title: Supplementary Data 2:
Description: Available information on the bacterial strains in which phiCjT23-like prophage area was detected with the locations of the putative prophage in the genome or genome contig
