# Supplementary material for: Cryo-EM structure of ssDNA bacteriophage ΦCjT23 provides insight into early virus evolution
Source: Nat Commun. 2022 Dec 3;13:7478. doi: 10.1038/s41467-022-35123-6 (PMC9719478; doi:10.1038/s41467-022-35123-6)
Supplement: Supplementary file 5 — Reporting Summary [file 41467_2022_35123_MOESM5_ESM.pdf]

nature portfolio

Corresponding author(s): Huiskonen, Juha T.

Last updated by author(s): Oct 10, 2022

Reporting Summary

Nature Portfolio wishes to improve the reproducibility of the work that we publish. This form provides structure for consistency and transparency in reporting. For further information on Nature Portfolio policies, see our Editorial Policies and the Editorial Policy Checklist.

Statistics

For all statistical analyses, confirm that the following items are present in the figure legend, table legend, main text, or Methods section.

n/a	Confirmed	
☐	☒	The exact sample size (*n*) for each experimental group/condition, given as a discrete number and unit of measurement
☐	☒	A statement on whether measurements were taken from distinct samples or whether the same sample was measured repeatedly
☒	☐	The statistical test(s) used AND whether they are one- or two-sided<br>*Only common tests should be described solely by name; describe more complex techniques in the Methods section.*
☒	☐	A description of all covariates tested
☒	☐	A description of any assumptions or corrections, such as tests of normality and adjustment for multiple comparisons
☐	☒	A full description of the statistical parameters including central tendency (e.g. means) or other basic estimates (e.g. regression coefficient) AND variation (e.g. standard deviation) or associated estimates of uncertainty (e.g. confidence intervals)
☒	☐	For null hypothesis testing, the test statistic (e.g. *F*, *t*, *r*) with confidence intervals, effect sizes, degrees of freedom and *P* value noted<br>*Give P values as exact values whenever suitable.*
☒	☐	For Bayesian analysis, information on the choice of priors and Markov chain Monte Carlo settings
☒	☐	For hierarchical and complex designs, identification of the appropriate level for tests and full reporting of outcomes
☒	☐	Estimates of effect sizes (e.g. Cohen's *d*, Pearson's *r*), indicating how they were calculated

Our web collection on statistics for biologists contains articles on many of the points above.

Software and code

Policy information about availability of computer code

Data collection	The cryo-EM data were collected using EPU software ver. 1.20.3.10 (Thermo Fisher Scientific)
Data analysis	CryoEM data analysis was performed with RELION 3.0 as part of Scipion 3.0. Coot 0.8.9.1 was used for model building. Phenix 1.19.2. was used for model refinement. Homologous Structure Finder software 1.1 was used to compare structures. ChimeraX 1.1.1 was used for making figures.

For manuscripts utilizing custom algorithms or software that are central to the research but not yet described in published literature, software must be made available to editors and reviewers. We strongly encourage code deposition in a community repository (e.g. GitHub). See the Nature Portfolio guidelines for submitting code & software for further information.

Data

Policy information about availability of data

All manuscripts must include a data availability statement. This statement should provide the following information, where applicable:

- Accession codes, unique identifiers, or web links for publicly available datasets
- A description of any restrictions on data availability
- For clinical datasets or third party data, please ensure that the statement adheres to our policy

The sequence data generated in this study have been deposited in the GenBank genetic sequence database under the accession codes ON067806 [https://www.ncbi.nlm.nih.gov/nuccore/ON067806] (ΦCjT23 genomic sequence) and CP097434 [https://www.ncbi.nlm.nih.gov/nuccore/CP097434] (Flavobacterium sp B183 whole genome sequence), The cryo-EM data have been deposited in the Electron Microscopy Data Bank (EMDB) under the accession codes EMD-15042

[https://www.ebi.ac.uk/emdb/entry/EMD-15042], EMD-15044 [https://www.ebi.ac.uk/emdb/entry/EMD-15044], EMD-15045 [https://www.ebi.ac.uk/emdb/entry/EMD-15045], EMD-15046 [https://www.ebi.ac.uk/emdb/entry/EMD-15046], EMD-15047 [https://www.ebi.ac.uk/emdb/entry/EMD-15047], EMD-15048 [https://www.ebi.ac.uk/emdb/entry/EMD-15048], EMD-15049 [https://www.ebi.ac.uk/emdb/entry/EMD-15049] EMD-15050 [https://www.ebi.ac.uk/emdb/entry/EMD-15050], EMD-15051 [https://www.ebi.ac.uk/emdb/entry/EMD-15051] (cryo-EM density maps) and in the Protein Data Bank (PDB) under the accession codes 7ZZZ [http://doi.org/10.2210/pdb7ZZZ/pdb], 8A01 [http://doi.org/10.2210/pdb8A01/pdb], 8A02 [http://doi.org/10.2210/pdb8A02/pdb], 8A03 [http://doi.org/10.2210/pdb8A03/pdb], 8A04 [http://doi.org/10.2210/pdb8A04/pdb], 8A05 [http://doi.org/10.2210/pdb8A05/pdb], 8A06 [http://doi.org/10.2210/pdb8A06/pdb] (atomic models). All other data generated or analysed during this study are included in this published article (and its supplementary information files). Source data are provided with this paper.

Human research participants

Policy information about studies involving human research participants and Sex and Gender in Research.

Reporting on sex and gender	N/A
Population characteristics	N/A
Recruitment	N/A
Ethics oversight	N/A

Note that full information on the approval of the study protocol must also be provided in the manuscript.

Field-specific reporting

Please select the one below that is the best fit for your research. If you are not sure, read the appropriate sections before making your selection.

[x] Life sciences [ ] Behavioural & social sciences [ ] Ecological, evolutionary & environmental sciences

For a reference copy of the document with all sections, see nature.com/documents/nr-reporting-summary-flat.pdf

Life sciences study design

All studies must disclose on these points even when the disclosure is negative.

Sample size	Sufficient sample size (number of cryoEM images and extracted particles) was determined by collecting enough data that would result a reconstructed map at sufficient resolution (estimated by Fourier shell correlation). Adsorption assay was done in three replicates.
Data exclusions	Micrographs that failed in contrast transfer function estimation were discarded. Particles that did not show features of the capsid were discarded in 2D-classification step.
Replication	The data were divided in two random subsets and processed separately as part of RELION's gold standard refinement protocol. This workflow produces the same result every time. Also for other types of experiments, all attempts at replication were successful.
Randomization	CryoEM data were randomly split in two half data sets in the beginning of the image processing (gold standard refinement). Other type of randomisation was not relevant. Randomization was not relevant for adsorption assays.
Blinding	Blinding was not used as samplings, data collection and data analysis were done in an unbiased way.

Reporting for specific materials, systems and methods

We require information from authors about some types of materials, experimental systems and methods used in many studies. Here, indicate whether each material, system or method listed is relevant to your study. If you are not sure if a list item applies to your research, read the appropriate section before selecting a response.

Materials & experimental systems

n/a	Involved in the study	
[x]	[ ]	Antibodies
[x]	[ ]	Eukaryotic cell lines
[x]	[ ]	Palaeontology and archaeology
[x]	[ ]	Animals and other organisms
[x]	[ ]	Clinical data
[x]	[ ]	Dual use research of concern

Methods

n/a	Involved in the study	
[x]	[ ]	ChIP-seq
[x]	[ ]	Flow cytometry
[x]	[ ]	MRI-based neuroimaging
